# The development and application of a high-sensitivity immunoassay for cardiac myosin–binding protein C

**DOI:** 10.1016/j.trsl.2015.11.008

**Published:** 2016-04

**Authors:** Jack Marjot, Christoph Liebetrau, Robert J. Goodson, Thomas Kaier, Ekkehard Weber, Peter Heseltine, Michael S. Marber

**Affiliations:** a King's College London BHF Centre, The Rayne Institute, St Thomas' Hospital, London, UK; b Kerckhoff Heart and Thorax Center, Department of Cardiology, Bad Nauheim, Germany; c DZHK (German Centre for Cardiovascular Research), Partner Site RheinMain, Bad Nauheim, Germany; d Singulex Inc, Alameda, Calif; e Institute of Physiological Chemistry, Martin Luther University Halle-Wittenberg, Halle, Germany

**Keywords:** ACS, acute coronary syndrome, AMI, acute myocardial infarction, cMyC, cardiac myosin–binding protein C, cTn, cardiac troponin, CV, coefficient of variation, DE, detected event, LoB, limit of blank, LoD, lower limit of detection, LLoQ, lower limit of quantification, MP, magnetic microparticle, NSTE-ACS, non–ST-elevation acute coronary syndrome

## Abstract

Cardiac troponins (cTns) are released and cleared slowly after myocardial injury. Cardiac myosin–binding protein C (cMyC) is a similar cardiac-restricted protein that has more rapid release and clearance kinetics. Direct comparisons are hampered by the lack of an assay for cMyC that matches the sensitivity of the contemporary assays for cTnI and cTnT. Using a novel pair of monoclonal antibodies, we generated a sensitive assay for MyC on the Erenna platform (Singulex) and compared serum concentrations with those of cTnI (Abbott) and cTnT (Roche) in stable ambulatory cardiac patients without evidence of acute cardiac injury or significant coronary artery stenoses. The assay for cMyC had a lower limit of detection of 0.4 ng/L, a lower limit of quantification (LLoQ) of 1.2 ng/L (LLoQ at 20% coefficient of variation [CV]) and reasonable recovery (107.1 ± 3.7%; mean ± standard deviation), dilutional linearity (101.0 ± 7.7%), and intraseries precision (CV, 11 ± 3%) and interseries precision (CV, 13 ± 3%). In 360 stable patients, cMyC was quantifiable in 359 patients and compared with cTnT and cTnI measured using contemporary high-sensitivity assays. cMyC concentration (median, 12.2 ng/L; interquartile range [IQR], 7.9–21.2 ng/L) was linearly correlated with those for cTnT (median, <3.0 ng/L; IQR, <3.0–4.9 ng/L; R = 0.56, *P* < 0.01) and cTnI (median, 2.10 ng/L; IQR, 1.3–4.2 ng/L; R = 0.77, *P* < 0.01) and showed similar dependencies on age, renal function, and left ventricular function. We have developed a high-sensitivity assay for cMyC. Concentrations of cMyC in clinically stable patients are highly correlated with those of cTnT and cTnI. This high correlation may enable ratiometric comparisons between biomarkers to distinguish clinical instability.

**At a Glance Commentary**

Marjot J, et al.

**Background**

Cardiac myosin–binding protein C (cMyC) is a protein with cardiac-restricted expression that we have previously shown appears in the systemic circulation after acute myocardial injury using a relatively insensitive assay. This article describes a high-sensitivity assay for cMyC, which demonstrates that it can be measured at baseline in almost all individuals, and in a stable population its concentration correlates with those for cTnI and cTnT.

**Translational Significance**

This article acts as the foundation for a study using the assay described here in patients presenting with suspected acute myocardial infarction to compare the diagnostic and prognostic performances of cMyC with cTnT and cTnI.

## Introduction

Acute myocardial infarction (AMI) carries a poor prognosis that can be improved by timely intervention. It must therefore be rapidly identified and differentiated from other causes of chest pain.^1^ Cardiac necrosis biomarkers have become crucial in affirming or excluding AMI in suspected non–ST-elevation acute coronary syndromes (NSTE-ACSs) and are needed to confirm the diagnosis in an appropriate clinical context.^2^ Cardiac troponins (cTns) have emerged as the gold standard and are incorporated in the universal definition of AMI.^2^ However, the cTns have potential drawbacks and new necrosis biomarkers could prove invaluable.^3^

The concentration of cTn rises slowly after acute myocardial injury and does not peak until 16–18 hours after the onset of chest pain.^4^ To triage and treat NSTE-ACS early, it is therefore necessary to heed cTn concentrations close to the 99th percentile of a healthy population.^5^ However, triage is confounded by the assays' decreased specificity for myocardial infarction when used in this way. In addition, diagnostic sensitivity may also be poor because up to 25% of patients with an eventual diagnosis of AMI are less than this threshold at presentation.^6^ Furthermore, although initial reports suggested that these assays allow more rapid diagnosis of AMI when the event is defined by a classic cTn assay,^7^, ^8^ this advantage is probably lost when contemporary high-sensitivity assays are also used to define the index event.^9^ These drawbacks are acknowledged in the recently updated guidelines for the management of NSTE-ACSs that adopt cutoffs substantially less than the 99th percentile to “rule-out” AMI and substantially greater than the 99th percentile to “rule-in” AMI.^10^ This improves sensitivity and specificity at the expense of increasing the number of patients with indeterminate troponins requiring further observation and increased testing.

The sarcomeric protein, cardiac myosin–binding protein C (C-protein, MYBPC3, cMyBP-C, or cMyC), is abundant^11^ and released rapidly into the coronary effluent.^12^ Recently, we demonstrated that cMyC accumulates more rapidly in the serum than cTnT; using timed iatrogenic injury in the setting of alcohol septal ablation for hypertrophic cardiomyopathy.^13^ Although after coronary artery bypass surgery, cMyC disappeared more rapidly than cTnT.^13^ However, comparisons were hindered by an insensitive assay for cMyC (lower limit of quantification [LLoQ], 80 ng/L), which consequently could only be quantified after injury had occurred. Without a sensitive assay for cMyC it is not possible to compare its diagnostic performance for AMI in suspected NSTE-ACS with those of cTnI and cTnT. The purpose of this study was to create and validate such a high-sensitivity assay.

## Materials and Methods

### Immunoassay for cMyC

We have previously described the creation, biophysical selection, and organ specificity of mouse monoclonal antibodies recognizing cardiac-restricted epitopes within the N-terminus of cMyC.^13^ Two of these antibodies, 1A4 and 3H8, were used to create a sensitive sandwich immunoassay. Subsequently, we describe the optimized assay on the Erenna platform (Merck KGaA, Darmstadt, Germany).

Magnetic microparticles (MPs; Singulex) for capture were prepared by binding 25 μg of mouse monoclonal (1A4) per milligram of MPs. The coated MPs were diluted in assay buffer (Singulex proprietary mix with custom 450 mM NaCl and 0.5% Triton X-100) to 100 μg/mL. Serum, plasma, or analyte (recombinant C0C2 domain of cMyC^13^) was diluted 1:1 in an equal volume of standard diluent (Singulex) and 100 μL added per well of a 96-well assay plate. Samples or standards were then exposed to 100 μL of coated MPs and agitated for 2 hours at 25°C. MPs were retained via a magnetic bed with unbound material removed in a single wash step. Fluorescently labeled mouse monoclonal (3H8) detection antibody was diluted in assay buffer (Singulex) to 100 ng/mL. To each well, 20 μL of detection antibody was added and the MPs agitated for 1 hour at 25°C, retained via a magnetic bed, and then washed 4 times to remove any unbound detection reagent. The MPs were then transferred to a new plate and all buffer was aspirated. The MPs were then exposed to 20 μL/well of elution buffer B (Singulex) for 5 minutes at 25°C before transferring to a 384-well plate containing 10 μL/well of neutralization buffer D (Singulex). Fluorescent label was then detected by single molecule counting using the Erenna system (Singulex) with a dwell time of 60 s per well. Three signal outputs were obtained from the Erenna System: detected events (DEs; low end signal), event photons (low end and higher end signal), and total photons (high end signal).

### Assessing assay performance under serum-free conditions

Having established a refined set of assay conditions, assay performance was assessed using a 12-point standard curve. Each point consisted of three 3-fold serially diluted cMyC concentrations to S4, followed by seven 2-fold serial dilutions to S11. All dilutions were in standard diluent (Singulex). The curve ranged from 0.58 to 2000 ng/L (S1–S11) with a 0 ng/L anchor of unadulterated standard diluent (Singulex). The lower limit of detection (LoD) was defined as 2.5 × standard deviation background divided by slope, and the LLoQ was defined as the lowest point on the standard curve, which has a coefficient of variation (CV) ≤20% where the back interpolated concentration had a recovery percent bias ≤20%.^14^

### Assay verification in human serum and plasma

Interassay and intra-assay series precision was evaluated in human serum samples that were tested unadulterated and spiked with 200 ng/L of cMyC. The samples were diluted 2-fold in standard diluent (1:1 mix) before assaying 6 replicates per sample on Day 1 and 3 replicates per sample on Day 2. Spike recovery was calculated by subtracting the dilution-corrected endogenous cMyC concentration from the dilution-corrected spiked value divided by the expected value. Dilutional linearity was evaluated by serial dilution of spiked human plasma. Linearity was calculated by dividing the dilution-corrected cMyC concentration by the preceding value, expressed as a percentage.

### MyC concentrations in human serum

Between July 2009 and January 2014, 5329 patients were referred to the Kerckhoff Heart and Thorax Center for elective coronary angiography and provided written informed consent for their participation in blood-based biomarker studies as per institutional ethics board (FF 43/2010). The research was carried out according to the Code of Ethics of the World Medical Association (Declaration of Helsinki), informed consent was obtained, and the author's institutional review board has approved the study.

From this population we selected 360 serum samples based on the absence of obstructive stenoses (<50%) on invasive coronary angiography and normal (<14 ng/L) high-sensitivity cTnT, renal function, and liver function. Further criteria used to choose samples were prior measurement of high-sensitivity cTnI and sufficient volume of stored serum to allow duplicate measurements of cMyC (>100 μL). cTnT was measured in serum with the high-sensitivity electrochemiluminescence immunoassay (Elecsys Analyzer 2010; Roche Diagnostics, Mannheim, Germany). For the cTnT assay, the limit of blank (LoB) = 3.0 ng/L, LoD = 5.0 ng/L, and LLoQ = 13.0 ng/L. The lowest concentration measurable with a CV <10% for this assay is 13.5 ng/L. The recommended clinical decision limit (99th percentile) for rule out of AMI using this assay is 14.0 ng/L. Concentrations of cTnT less than 3 ng/L (LoB) were not returned and therefore assigned a value of 1.5 ng/L in all analyses.

cTnI was measured in serum with the high-sensitivity chemiluminescent immunoassay (ARCHITECT STAT High Sensitive Troponin; Abbott Laboratories, Abbott Park, Illinois). For the cTnI assay, LoD = 1.2 ng/L and LLoQ = 4.7 ng/L at a CV <10%. The 99th percentile is 15.6 ng/L in women and 34.2 ng/L in men. Concentrations less than the LoD were returned and used for comparisons, because all were greater than the locally determined LoB.

### Statistical analysis

The methods used to calculate the LoDs and LLoQs for MyC are described previously.

The Kolmogorov-Smirnov and Shapiro-Wilk tests were used to test if cMyC, cTnT, and cTnI concentrations were distributed normally. Spearman's rank test was used to assess correlation between the serum concentrations of each biomarker and to correlate the concentration of each marker to the continuous demographic variables of the sample population. Differences in the distribution of biomarker concentration across categories of dichotomous variables were examined using the independent-samples Mann-Whitney test. Stepwise multiple logistic regression analysis was used to assess independent association between the variables and biomarker concentrations. All analyses were carried out using SPSS v22. Normally distributed data are presented as the mean ± standard deviation. Statistical significance was set at *P* < 0.05.

## Results

### Analytic sensitivity of the cMyC assay

The 12-point calibration using recombinant C0C2 domain of cMyC in standard diluent is shown in Supplementary Table I. The DE counts are shown for serial dilutions >5. The linear regression relationship for S5–S12 is DE = 32.7 × [cMyC] + 46.1 (R^2^ = 0.9995), where [MyC] is in nanograms per liter. The LLoQ is 1.2 ng/L and the calculated LoD is 0.4 ng/L.

### Interseries and intraseries precision of the MyC assay in serum

Sera from 5 individuals were spiked with 200 ng/L of cMyC and subjected to repeated measurement. Six repeated measurements were made on Day 1 and 3 on Day 2. Supplementary Table II shows the CVs within and between assays. The average CV within assays was 11 ± 3% and between assays was 13 ± 3%.

### Analyte recovery from human serum and plasma

Supplementary Table III shows analyte recovery from serum and plasma samples of different individuals each spiked with 200 ng/L of recombinant cMyC. The recovery in serum was 108.0 ± 6.2% (excluding lipemic sample, 115.4 ± 15.8% with this sample included) and in plasma 107.1 ± 3.7%.

Dilutional linearity was tested using the finalized assay in plasma (see Supplementary Table IV). The results showed more than a 16-fold range of dilutions in plasma from 8 individuals, and linearity was 101 ± 7%.

### Comparison of cMyC, cTnT, and cTnI concentrations in stable patients

The demographics of the population cohort used to compare biomarker concentrations are shown in Table I.

**Table I:** Demographics of the patient population (N = 359 unless otherwise specified)

Demographic	n (%)
Male	146 (40.6%)
Current smoker	131 (36.4%)
BMI ≥ 30	124 (34.4%) [n = 358]
Diabetes	39 (10.8%)
Hyperlipidemia	181 (50.3%)
Family history	106 (29.4%)
β-Blocker	182 (50.6%)
Statin	82 (22.8%)
ACE-I/ARB	196 (54.4%)
Aspirin	159 (44.2%)
Digitalis	8 (2.2%)
Aldosterone antagonist	16 (4.4%)
Loop diuretic	56 (15.6%)
Thiazide diuretic	82 (22.8%)
COPD	24 (6.7%)
PVD	10 (2.8%)
Pulmonary HTN	7 (1.9%)
Angina	180 (50.0%)
AF/PPM	32 (8.9%) [n = 357]
	Mean (standard deviation)
Age (y)	60.0 (12.0)
BMI	29.0 (5.6) [n = 358]
GFR (mL/min/1.73 m^2^)	100.3 (25.7) [n = 352]
Creatinine (mg/dL)	0.8 (0.2) [n = 351]
LVEF (%)	53.7 (12.3) [n = 189]
Systolic BP (mm Hg)	134.5 (20.9) [n = 357]
[MyC] (ng/L)	17.6 (16.4)
[TnT] (ng/L)	3.4 (3.2)
[TnI] (ng/L)	3.5 (4.3)

*Abbreviations: ACE-I*, angiotensin-converting enzyme inhibitor; *AF*, atrial fibrillation; *ARB*, angiotensin receptor blocker; *BMI*, body mass index; *BP*, blood pressure; *COPD*, chronic obstructive pulmonary disease; *GFR*, glomerular filtration rate; *HTN*, hypertension; *LVEF*, left ventricular ejection fraction; *PPM*, permanent pacemaker; *PVD*, peripheral vascular disease.

Angina was defined as any symptom severity using Canadian Cardiovascular Society grades 1–4. Family history defined as a first degree relative with history of coronary artery disease and/or acute myocardial infarction and/or percutaneous coronary intervention and/or coronary artery bypass graft surgery.

**Fig 1:**
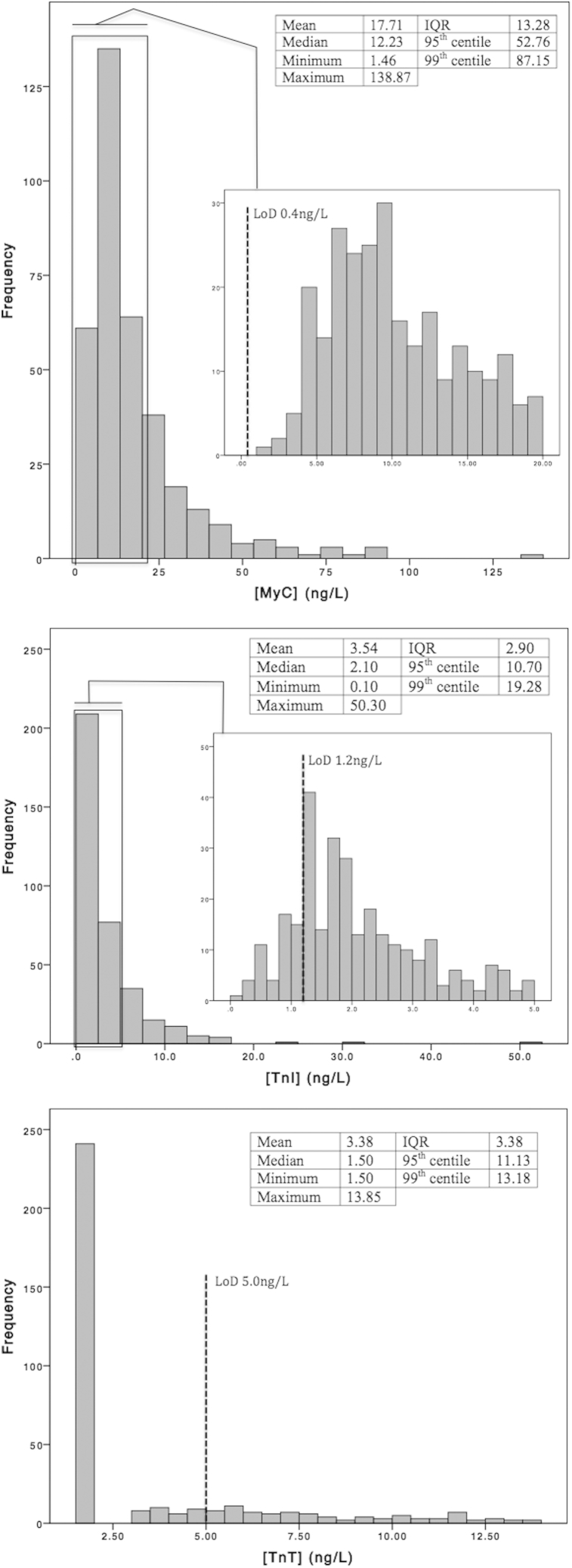
Distribution of cMyC, cTnI, and cTnT concentrations among 359 patients referred for elective coronary angiography with a cTnT <14 ng/L. To validate the cMyC assay described in Supplementary Table I, Supplementary Table II, Supplementary Table III, Supplementary Table IV we examined a stable patient cohort without acute myocardial injury. Also excluding acute myocardial injury by their mode of presentation, only patients with a [cTnT] less than the 99th percentile of a healthy “normal” population (14 ng/L) were included. All patients had a [cMyC] > LLoQ. Unfortunately, 274 patients (more than half the cohort) had a [cTnT] <5.0 ng/L, the LoD of the assay. Sera with a [cTnT] <3.0 ng/L (LoB) were assigned a value of 1.5 ng/L. Similarly, 52 patients had a [TnI] <1.2 ng/L, the LoD of the assay. For TnI the sera retained the value assigned by the assay because values were greater than the locally determined LoB. The differential sensitivities of the assay are the cause for artifactual distortion of low concentration portion of the histograms. Inset in each panel are the descriptors of the biomarker concentration distribution. None of the biomarkers are normally distributed. cMyC, cardiac myosin–binding protein C; cTn, cardiac troponin; IQR, interquartile range; LLoQ, lower limit of quantification; LoD, lower limit of detection.

Three hundred sixty serum samples with [cTnT] <14 ng/L were analyzed. In one of these samples, cMyC was less than the LLoQ. Our subsequent analysis was of the 359 patients with an evaluable cMyC. Of these 274 patients had cTnT (<5 ng/L), and 52 patients cTnI (<1.2 ng/L), concentrations less than the LoD. The resulting truncation of the leftmost portion of the concentration-frequency distribution is therefore evident for cTnT and cTnI but not for cMyC (see Fig 1). None of the concentration-frequency histograms were normally distributed. The summary statistics describing their distribution are inset in the respective panels of Fig 1. In absolute terms cMyC is approximately 5 times more abundant than either cTnI or cTnT, as previously noted.^13^ Our cMyC assay therefore has sensitivity at least as good as the current commercial assays for cTnT and cTnI. The question is whether the concentrations of cMyC are related to those of the cTns?

**Fig 2:**
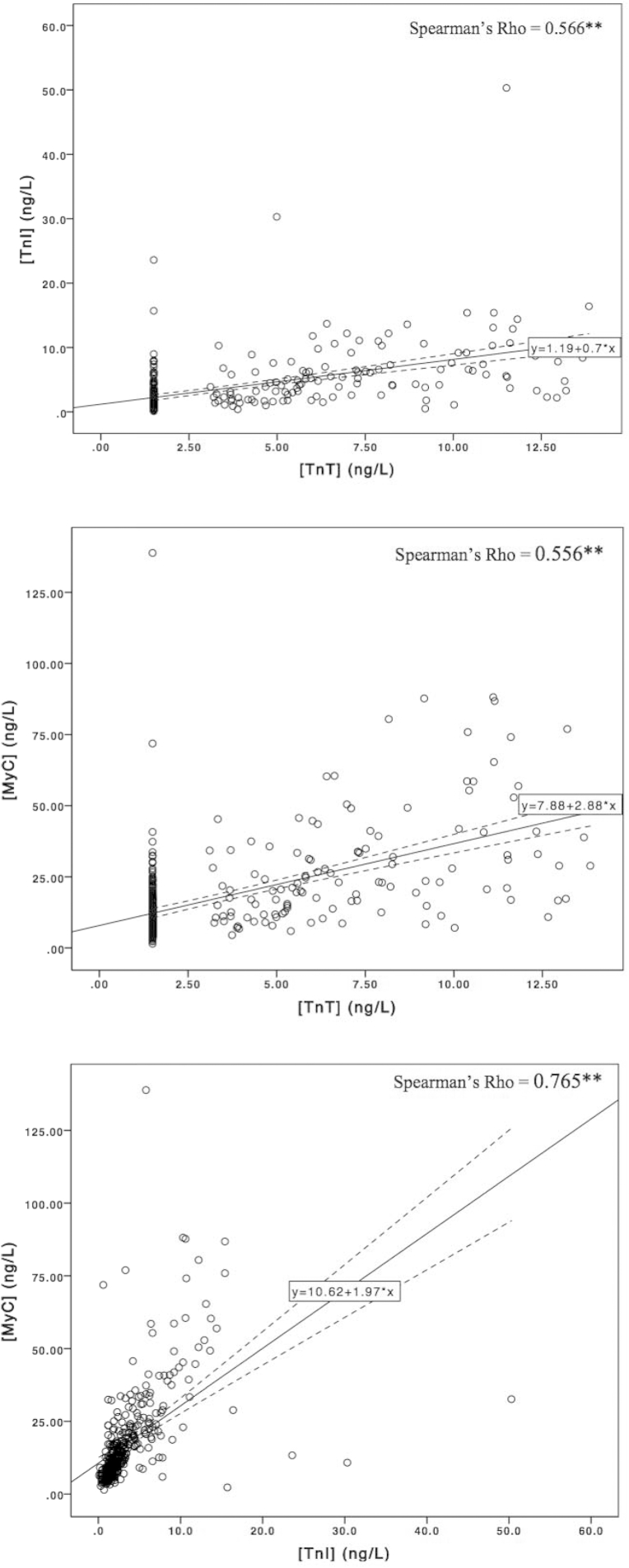
Relationships between cMyC, cTnI, and cTnT. All 3 biomarkers significantly correlate with one another. The correlation coefficient (Spearman's Rho) is shown on the upper right quadrant. ^∗∗^*P* < 0.01. cMyC, cardiac myosin–binding protein C; cTn, cardiac troponin.

Fig 2 shows the relationships between the biomarkers. Serum concentrations of cMyC, cTnT, and cTnI are all positively correlated with one another with the strongest association between [MyC] and [TnI].

Because the biomarkers are co-correlated we looked at the demographic variables known to influence [cTnT] and [cTnI] to determine if they similarly influence cMyC. The continuous variables are shown in Table II as correlation coefficients and the discontinuous variables in Table III as differences in mean biomarker concentration between those with and without the demographic feature. Generally, each of the biomarkers segregates similarly and concentrations are greater in patients with comorbidities.

**Table II:** Correlation coefficients (Spearman's Rho) between serum concentration of cTnI, cTnT, and cMyC and continuous variables in the sample population

Demographic variable	cTnI	cTnT	cMyC
Age	0.336∗	0.448∗	0.385∗
GFR	−0.224∗	−0.256∗	−0.288∗
Creatinine	0.197∗	0.220∗	0.284∗
LVEF	−0.208∗	−0.169†	−0.218∗
Systolic BP	0.116†	0.176∗	0.134†
BMI	0.069	0.068	0.011

*Abbreviations: BMI*, body mass index; *BP*, blood pressure; *cMyC*, cardiac myosin–binding protein C; *cTn*, cardiac troponin; *GFR*, glomerular filtration rate; *LVEF*, left ventricular ejection fraction.

∗ *P* < 0.01.

† *P* < 0.05.

**Table III:** Mean biomarker concentration in the each category of dichotomous population variable

Demographic variable	Mean concentration in each group (difference in mean concentrations)		
	cTnI (ng/L)	cTnT (ng/L)	cMyC (ng/L)
Nonmodifiable risk factors			
Family history vs no family history	3.27 vs 3.66 (0.38∗)	3.37 vs 3.38 (0.01)	15.0 vs 18.7 (3.71∗)
Female vs male	3.22 vs 3.69 (0.47)	3.27 vs 3.49 (0.22)	16.3 vs 19.4 (3.15)
Lifestyle			
Current smoker vs nonsmoker	3.12 vs 3.79 (0.67)	2.89 vs 3.66 (0.78∗)	16.0 vs 18.5 (2.52)
BMI ≥ 30 vs BMI < 30	4.07 vs 3.25 (0.82)	3.48 vs 3.30 (0.18)	16.2 vs 18.2 (2.04)
Comorbidities			
Pulmonary HTN vs no pulmonary HTN	8.84 vs 3.44 (5.41†)	8.61 vs 3.28 (5.33†)	44.4 vs 17.0 (27.32†)
AF/PPM vs sinus rhythm	5.88 vs 3.27 (2.61†)	4.92 vs 3.20 (1.71†)	27.0 vs 16.5 (10.55†)
Diabetes vs no diabetes	3.59 vs 3.54 (0.05)	4.21 vs 3.28 (0.93)	19.7 vs 17.3 (2.38)
Hyperlipidemia vs no hyperlipidemia	3.19 vs 3.90 (0.71)	3.07 vs 3.69 (0.62)	16.6 vs 18.6 (1.94)
COPD vs not COPD	3.98 vs 3.51 (0.47)	3.88 vs 3.34 (0.53)	17.3 vs 17.6 (0.38)
PVD vs no PVD	4.19 vs 3.53 (0.67)	3.37 vs 3.38 (0.01)	20.9 vs 17.5 (3.44)
Angina vs no angina	3.14 vs 3.95 (0.80)	3.07 vs 3.69 (0.62)	16.0 vs 19.2 (3.17)
Pharmacotherapy			
β-blocker vs no β-blocker	3.71 vs 3.37 (0.34)	3.52 vs 3.24 (0.28)	20.8 vs 14.3 (6.51†)
ACE-I/ARB vs no ACE-I/ARB	4.26 vs 2.68 (1.58†)	3.98 vs 2.66 (1.32†)	20.2 vs 14.5 (5.75†)
Aspirin vs no aspirin	3.13 vs 3.87 (0.75†)	2.98 vs 3.70 (0.72∗)	15.9 vs 19.0 (3.09∗)
Digitalis vs no digitalis	5.16 vs 3.51 (1.66)	5.60 vs 3.33 (2.27∗)	28.1 vs 17.4 (10.77†)
Aldosterone antagonist vs no aldosterone antagonist	5.37 vs 3.46 (1.91†)	5.86 vs 3.26 (2.59†)	30.5 vs 17.0 (13.52†)
Loop diuretic vs no loop diuretic	5.61 vs 3.16 (2.45†)	4.93 vs 3.09 (1.83†)	26.9 vs 15.9 (10.98†)
Thiazide diuretics vs no thiazide diuretic	4.56 vs 3.24 (1.32†)	4.39 vs 3.08 (1.30†)	23.1 vs 16.0 (7.16†)
Statin vs no statin	2.82 vs 3.76 (0.94∗)	2.70 vs 3.58 (0.88)	15.0 vs 18.3 (3.32)

*Abbreviations: ACE-I*, angiotensin-converting enzyme inhibitor; *AF*, atrial fibrillation; *ARB*, angiotensin receptor blocker; *BMI*, body mass index; *cMyC*, cardiac myosin–binding protein C; *COPD*, chronic obstructive pulmonary disease; *cTn*, cardiac troponin; *HTN*, hypertension; *PPM*, permanent pacemaker; *PVD*, peripheral vascular disease.

In brackets is the difference in mean biomarker concentrations between the 2 categories.

∗ *P* < 0.05.

† *P* < 0.01.

A stepwise multiple logistic regression analysis was performed to determine which independent variables statistically significantly predicted the serum biomarker concentrations, independently of other covariates (Table IV). In this analysis, age, gender, creatinine, pulmonary hypertension, and use of statins, loop diuretics, and β-blockers all statistically predicted cMyC (*P* < 0.05), R^2^ = 0.198, n = 346. Because left ventricular ejection fraction (LVEF) was only known for 189 samples, LVEF was omitted from the analysis to preserve sample size and statistical power. With LVEF included in the analysis, only creatinine, LVEF, and age significantly predicted cMyC (*P* < 0.01), R^2^ = 0.22, n = 183 (see Supplementary Table I, Supplementary Table II, Supplementary Table III, Supplementary Table IV, Supplementary Table V, Supplementary Table VI). The same model was applied to cTnI and cTnT, excluding LVEF as a variable. cTnI was significantly predicted by age, gender, use of angiotensin-converting enzyme inhibitor and, or angiotensin receptor blockers (ACE-I/ARBs), statins, and loop diuretics, and irregular or paced cardiac rhythm (*P* < 0.05), R^2^ = 0.153, n = 346. cTnT was significantly predicted by age, gender, family history of heart disease (see Table I for definition), pulmonary hypertension, angina, diabetes, and use of ACE-I/ARBs, aldosterone antagonists and statins (*P* < 0.05), R^2^ = 0.299, n = 346. A distinctive feature of MyC was its association with β-blocker use.

**Table IV:** Unstandardized coefficients (B) for those variables which independently predict biomarker concentration in stepwise linear multiple regression analysis (n = 346)

Demographic variable	Unstandardized coefficients (B)	Significance
MyC		
Creatinine	10.689	0.024
Age	0.286	0.000
Female	−5.128	0.004
Loop diuretic	6.889	0.002
Statin	−5.510	0.003
B-blocker	4.436	0.009
Pulmonary hypertension	14.813	0.024
TnI		
Age	0.067	0.000
Female	−1.879	0.000
ACE-I/ARB	1.136	0.014
Loop diuretic	1.639	0.010
Statin	−1.377	0.008
AF/PPM	1.572	0.047
TnT		
Age	0.114	0.000
Female	−1.012	0.001
ACE-I/ARB	0.825	0.007
Statin	−1.477	0.000
Aldosterone antagonist	2.001	0.007
Family history	0.820	0.013
Pulmonary hypertension	4.771	0.000
Angina	−0.603	0.040
Diabetes	0.934	0.047

*Abbreviations: ACE-I*, angiotensin-converting enzyme inhibitor; *AF*, atrial fibrillation; *ARB*, angiotensin receptor blocker; *MyC*, myosin-binding protein C; *PPM*, permanent pacemaker.

## Discussion

We have developed a high-sensitivity assay to measure cMyC in serum or plasma. In 360 stable patients with a cTnT <14 ng/L, cMyC was quantifiable in 359 patients, a sensitivity much greater than that achieved with cTnT (4 patients with greater than LLoQ = 13.0 ng/L) or cTnI (78 patients with greater than LLoQ = 4.7 ng/L). cMyC is the first cardiac-specific marker of injury to be described since cTnT and cTnI. Generally, concentrations of cMyC were highly correlated with those of cTnI and cTnT and were influenced by the same demographic features including gender, age, renal function, left ventricular function, medication, and heart rhythm.

The close correlation between cMyC and cTnT/cTnI is surprising because their locations within the sarcomere differ.^15^ cTnT and cTnI are adjacent proteins on the thin filament (actin), whereas cMyC, as its name suggests, is predominantly bound to the thick filament (myosin). The precise reason for the appearance of cardiac sarcomeric proteins in the peripheral blood of healthy individuals is not known. However, because none of these proteins are actively exported, and an intact sarcolemma is impervious to proteins >40 kDa,^16^ their appearance in the circulation most likely represents “stable” slow attrition and dissolution of cardiac myocytes. In such a scenario, the release rates of all cardiac-specific proteins are likely to co-correlate because they document the same fundamental process. Furthermore, the rapidity of this process is likely to be influenced by traditional cardiac risk factors explaining the correlation with gender and age, whereas the progression of this process will be documented by other measures of cardiac injury explaining the correlation with left ventricular function, pulmonary artery hypertension, and medication. The correlation with renal function is likely to have more complex explanations including the renal excretion of immunoreactive N-terminal fragments of cMyC, cTnI, and cTnT; the accumulation of waste products that increase the rate of myocyte attrition; or common factors that cause cellular injury to both the heart and the kidney.

Although, the factors affecting cTnI, cTnT, and cMyC are broadly very similar (see Table II, Table III), serum cMyC concentration is particularly affected by β-blocker prescription with an average 6.5 ng/L higher concentration in those taking medications of this class (a relationship that continues to be significant after multiple regression analysis). A possible explanation for this exceptional dichotomy between the biomarkers may relate to protein kinase A–dependent phosphorylation of critical serine residues within the M domain of cMyC.^17^ When phosphorylated, these residues more effectively guard a calpain cleavage site within cMyC.^15^, ^17^ Cleavage at this site releases a 40 kDa N-terminal fragment, the dominant fragment we observed in serum of patients with AMI.^12^, ^13^, ^18^ Interestingly this fragment may act as a “poison peptide” causing cardiac dysfunction.^15^ Thus, unlike cTnI and cTnT, cMyC may not just be a bystander biomarker of cardiac injury, but lie on the causal pathway leading to myocardial disease.

Our ultimate aim is to determine if cMyC is a “better” diagnostic biomarker of acute myocardial injury than cTnT or cTnI. On the basis of our previous findings with a much less sensitive assay, after iatrogenic myocardial injury cMyC is released and cleared more rapidly from the peripheral circulation than cTnT.^13^ The findings presented here are necessary stepping stones toward a large study of patients with suspected NSTE-ACS where the diagnostic utility of cMyC can be compared with cTnI and cTnT. Nonetheless, it is tempting to speculate how our present study will have impact on the diagnostic performance of cMyC in this clinical scenario. We had hoped cMyC concentrations would not be influenced by age, gender, renal function, and other cardiac risk factors. Our results clearly suggest that baseline cMyC concentrations will be higher in those at risk of an NSTE-ACS than in healthy controls. Thus, it is likely cMyC will have the same inadequacy as cTnI and cTnT in differentiating chronic increases in biomarker concentration from the minor increases associated with the start of an acute myocardial injury event. However, if our findings of faster MyC release in iatrogenic injury hold true with spontaneous myocardial injury, then the close correlation between MyC and cTnI/cTnT could become uncoupled as the biomarkers rise asymmetrically during acute injury. On the other hand, the abundance, ease of measurement, and correlation of cMyC with comorbidities may provide an advantage in the monitoring of chronic disease.

One of the major limitations of the present study is that the study population was not healthy, and we therefore cannot estimate the 99th percentile concentration for cMyC. In choosing the population to validate our novel assay, we thought it more important to have a complete description of their demographics with the availability of other laboratory measures, including contemporary high-sensitivity cTnI and cTnT. This choice was consolidated by the lack of guidance on how rigorously to exclude covert cardiac disease in a healthy cohort and the influence this uncertainty has on the 99th percentile returned by that particular unique healthy cohort. Finally, we reasoned that the patient population we studied is more representative of those that will attend with a suspected NTSE-ACS event than a healthy younger cohort without cardiac risk factors. Nonetheless, the 99th percentiles returned in our population closely match those defined in healthy control populations for the assays we used for TnT (13 vs 14 ng/L, respectively) and TnI (19 vs 22 ng/L, respectively).

A further limitation is that the Erenna platform on which the cMyC assay is performed is currently only available for research purposes and cannot provide the flexibility or turn-around times required for clinical use. These deficiencies could be addressed by migration to another platform or through the development of the Erenna platform.

Another “high-sensitivity” assay for cMyC has been described recently.^19^ However, this assay has a sensitivity of 2–3 orders of magnitude lower than ours and reports a mean difference in cMyC concentration between controls and patients with AMI of approximately 3-fold (∼1.5 μg/L increasing to ∼5 μg/L).^19^ These concentrations are difficult to reconcile with those presented here or previously.^13^

## Conclusions

We have developed and validated a sensitive assay for cMyC, which for the first time allows this cardiac-specific marker of myocardial injury to be quantified in ambulatory patients. The diagnostic performance of this assay is yet to be compared with cTnI and cTnT in the setting of NSTE-ACS.

## Figures and Tables

**Supplementary Table I:** Analytic performance of the cMyC assay under serum-free conditions

Standard	Expected [cMyC], ng/L	n	Mean DE	Observed [cMyC], ng/L	%CV	Recovery
S1	2000	3		1998.55	3%	100%
S2	666.67	3		732.07	4%	110%
S3	222.22	3		227.84	12%	103%
S4	74.07	3		68.72	8%	93%
S5	37.04	3	1254	36.86	3%	100%
S6	18.52	3	665.33	19.63	4%	106%
S7	9.26	3	344.33	9.79	4%	106%
S8	4.63	2	189	4.7	23%	102%
S9	2.32	3	106.33	1.85	5%	80%
S10	1.16	2	89.5	1.27	13%	110%
S11	0.58	3	74	0.75	20%	129%
S12	0	3	51	ND	—	—

*Abbreviations: cMyC*, cardiac myosin–binding protein C; *CV*, coefficient of variation; *DE*, detected event; *ND*, not determined.

**Supplementary Table II:** Analytic precision of the cMyC assay using serum from 5 individuals with (+200) and without the addition of a 200 ng/L spike of recombinant cMyC

ID	Day 1						Intraseries			Day 2			Interseries		
	1	2	3	4	5	6	Mean	SD	%CV	1	2	3	Mean	SD	%CV
1	4.77	6.22	4.54	4.6	4.54	4.77	4.91	0.65	13%	4.97	—	6.35	5.09	0.75	15%
1 + 200	249.2	228.4	—	241.6	248.4	286.8	250.87	21.74	9%	223.9	234.1	214.7	240.88	22.1	9%
2	20.66	19.39	14.71	18.03	15.89	17.36	17.67	2.19	12%	24.79	20.82	26.69	19.81	3.95	20%
2 + 200	322.9	291.5	304.6	290.5	361	382	325.43	38.15	12%	274.1	311.4	246.9	309.44	41.8	14%
3	71.53	70.13	70.6	66.66	59.34	62.43	66.78	4.95	7%	64.44	55.36	51.45	63.55	7.07	11%
3 + 200	252	241.9	276.5	264.6	271.4	342.4	274.79	35.47	13%	252	261.3	229	265.68	32.3	12%
4	13.55	14.76	13.65	13.13	11.28	10.23	12.77	1.68	13%	16.97	17.07	12.76	13.71	2.3	17%
4 + 200	216.5	199.2	249.8	273.8	224.1	236.6	233.32	26.26	11%	197.5	203.4	220.4	224.57	25.3	11%
5	17.74	17.92	16.76	15.34	11.49	16.13	15.9	2.37	15%	17.66	17.62	13.49	16.02	2.23	14%
5 + 200	232.6	255.3	258.8	228	252.9	254.9	247.07	13.19	5%	187.8	204.8	216.2	232.36	25.4	11%

*Abbreviations: cMyC*, cardiac myosin–binding protein C; *CV*, coefficient of variation; *DE*, detected event.

All values are in nanograms per liter unless stated otherwise. The serum of individual number 2 was lipemic.

**Supplementary Table III:** Spike recovery in human serum and plasma. Samples are sera or plasma from different individuals with (+200) and without the addition of a 200 ng/L spike of recombinant cMyC

ID	n	Observed [cMyC], ng/L	SD	%CV	% Spike recovery
Serum					
1	8	5.09	0.75	15	
1 + 200	9	240.88	22.07	9	118
2	9	19.81	3.95	20	
2 + 200	9	309.44	41.79	14	145
3	9	63.55	7.07	11	
3 + 200	9	265.68	32.28	12	101
4	9	13.71	2.30	17	
4 + 200	9	224.57	25.27	11	105
5	9	16.02	2.23	14	
5 + 200	9	232.36	25.43	11	108
Plasma					
6	3	1.18	0.10	9	
6 + 200	3	206.14	3.18	2	102
7	3	6.23	0.13	2	
7 + 200	3	228.05	9.71	4	111
8	3	5.7	0.84	15	
8 + 200	3	221.06	7.70	3	108
9	3	3.98	0.22	6	
9 + 200	3	212.16	15.7	7	104
10	3	1.28	0.27	21	
10 + 200	3	227.78	10.4	5	113
11	3	1.2	0.21	17	
11 + 200	3	212.03	16.3	8	105
12	3	3.73	0.20	5	
12 + 200	3	212.46	2.43	1	104
13	3	10.29	0.41	4	
13 + 200	3	230.68	11.20	5	110

*Abbreviations: cMyC*, cardiac myosin–binding protein C; *CV*, coefficient of variation; *SD*, standard deviation.

All values are in nanograms per liter unless stated otherwise. The serum of individual number 2 was lipemic and excluded from analysis of average % spike recovery.

**Supplementary Table IV:** Dilutional linearity of human plasma spiked with 200 ng/L of cMyC

Sample	n	Dilution factor	Mean [cMyBP-C], pg/mL	SD	%CV	Dilution corrected [cMyBP-C], ng/L	Dilutional linearity
1	3	2	206.14	3.18	2%	412.28	
1	3	4	105.42	7.16	7%	421.68	102%
1	3	8	49.05	2.09	4%	392.43	93%
1	3	16	26.83	1.72	6%	429.25	109%
2	3	2	228.05	9.71	4%	456.09	
2	3	4	123.61	4.54	4%	494.43	108%
2	3	8	54.84	4.57	8%	438.72	89%
2	3	16	28.34	0.15	1%	453.43	103%
3	3	2	221.06	7.5	3%	442.12	
3	3	4	114.14	2.55	2%	456.56	103%
3	3	8	54.71	3.09	6%	437.67	96%
3	3	16	29.55	1.48	5%	472.87	108%
4	3	2	212.16	15.7	7%	424.33	
4	3	4	116.07	1.48	1%	464.26	109%
4	3	8	53.23	1.94	4%	425.81	92%
4	3	16	28.98	1.19	4%	463.61	109%
5	3	2	227.78	10.4	5%	455.56	
5	3	4	108.27	6.32	6%	433.06	95%
5	3	8	51.02	1.67	3%	408.2	94%
5	3	16	27.81	0.94	3%	445.03	109%
6	3	2	212.02	16.3	8%	424.03	
6	3	4	102.62	3.97	4%	410.47	97%
6	3	8	48.11	1.39	3%	384.84	94%
6	3	16	27.04	0.82	3%	432.69	112%
7	3	2	212.46	2.43	1%	424.91	
7	3	4	101.82	2.19	2%	407.28	96%
7	3	8	46.98	1.31	3%	375.83	92%
7	3	16	26.4	1.33	5%	422.35	112%
8	3	2	230.68	11.2	5%	461.36	
8	3	4	103.66	5.41	5%	414.65	90%
8	3	8	50.05	2.68	5%	400.37	97%
8	3	16	28	1.47	5%	447.96	112%

*Abbreviations: cMyC*, cardiac myosin–binding protein C; *CV*, coefficient of variation; *SD*, standard deviation.

**Supplementary Table V:** Results of stepwise multiple linear regression analysis for each biomarker, excluding LVEF as an independent variable (n = 346)

Demographic variable	Unstandardized coefficients		Standardized coefficients	*t*	Significance	95.0% Confidence interval for B	
	B	Standard error	Beta			Lower bound	Upper bound
MyC							
Creatinine	10.689	4.714	0.128	2.268	0.024	1.418	19.961
Age	0.286	0.068	0.218	4.197	0.000	0.152	0.420
Female	−5.128	1.774	−0.163	−2.890	0.004	−8.618	−1.638
Loop diuretic	6.889	2.200	0.160	3.132	0.002	2.562	11.216
Statin	−5.510	1.869	−0.149	−2.948	0.003	−9.187	−1.834
β-Blocker	4.436	1.679	0.142	2.641	0.009	1.132	7.739
Pulmonary HTN	14.813	6.547	0.113	2.263	0.024	1.935	27.690
TnI							
Age	0.067	0.019	0.186	3.552	0.000	0.030	0.103
Female	−1.879	0.444	−0.218	−4.235	0.000	−2.751	−1.006
ACE-I/ARB	1.136	0.461	0.132	2.467	0.014	0.230	2.042
Loop diuretic	1.639	0.633	0.139	2.589	0.010	0.394	2.885
Statin	−1.377	0.514	−0.136	−2.680	0.008	−2.388	−0.367
AF/PPM	1.572	0.790	0.105	1.990	0.047	0.018	3.126
TnT							
Age	0.114	0.013	0.431	8.889	0.000	0.088	0.139
Female	−1.012	0.300	−0.160	−3.373	0.001	−1.602	−0.422
Family history	0.820	0.326	0.119	2.510	0.013	0.177	1.462
Pulmonary HTN	4.771	1.223	0.180	3.901	0.000	2.365	7.176
Angina	−0.603	0.293	−0.096	−2.059	0.040	−1.179	−0.027
Diabetes	0.934	0.468	0.093	1.995	0.047	0.013	1.855
Statin	−1.477	0.348	−0.198	−4.244	0.000	−2.162	−0.793
ACE-I/ARB	0.825	0.302	0.130	2.727	0.007	0.230	1.420
Aldosterone antagonists	2.001	0.732	0.129	2.735	0.007	0.562	3.441

*Abbreviations: ACE*, angiotensin-converting enzyme inhibitor; *AF*, atrial fibrillation; *ARB*, angiotensin receptor blocker; *HTN*, hypertension; *LVEF*, left ventricular ejection fraction; *MyC*, myosin-binding protein C; *PPM*, permanent pacemaker.

**Supplementary Table VI:** Results of stepwise multiple linear regression analysis for each biomarker, including LVEF as an independent variable (n = 183)

Demographic variable	Unstandardized coefficients		Standardized coefficients	*t*	Significance	95.0% Confidence interval for B	
	B	Standard error	Beta			Lower bound	Upper bound
MyC							
Creatinine	23.452	6.034	0.261	3.886	0.000	11.545	35.360
LVEF	−0.366	0.094	−0.263	−3.915	0.000	−0.551	−0.182
Age	0.339	0.089	0.255	3.791	0.000	0.163	0.516
TnI							
Female	−2.772	0.687	−0.280	−4.035	0.000	−4.127	−1.416
LVEF	−0.106	0.028	−0.257	−3.744	0.000	−0.162	−0.050
Age	0.097	0.027	0.246	3.528	0.001	0.043	0.151
TnT							
Age	0.113	0.017	0.431	6.533	0.000	0.079	0.147
LVEF	−0.059	0.017	−0.216	−3.398	0.001	−0.094	−0.025
Pulmonary HTN	5.214	1.262	0.255	4.131	0.000	2.723	7.705
Statin	−1.797	0.531	−0.212	−3.382	0.001	−2.845	−0.748
Female	−1.401	0.417	−0.213	−3.361	0.001	−2.224	−0.578
Family history	1.130	0.479	0.149	2.361	0.019	0.185	2.074
ACE-I/ARB	1.036	0.440	0.150	2.358	0.020	0.169	1.904

*Abbreviations: ACE*, angiotensin-converting enzyme inhibitor; *ARB*, angiotensin receptor blocker; *HTN*, hypertension; *LVEF*, left ventricular ejection fraction; *MyC*, myosin-binding protein C.
